# First-Line Use of Vemurafenib to Enable Thyroidectomy and Radioactive Iodine Ablation for BRAF-Positive Metastatic Papillary Thyroid Carcinoma: A Case Report

**DOI:** 10.1177/2324709615603723

**Published:** 2015-09-03

**Authors:** Bao D. Dao, Ildiko Lingvay, Joseph Sailors, Michael Landay, Gabriel Shapiro

**Affiliations:** 1 University of Texas Southwestern Medical Center, Dallas, TX, USA

**Keywords:** BRAF mutation, BRAF inhibitor, vemurafenib, papillary thyroid carcinoma, differentiated thyroid carcinoma

## Abstract

*Background*. Patients with metastatic or radioactive iodine refractory papillary thyroid carcinoma (PTC) have poor prognosis due to ineffective therapy for this condition beyond surgery and radioactive iodine (RAI or ^131^I). BRAF mutation occurs in more than 44% of PCT. Tyrosine kinase inhibitors, the most commonly used agents for these patients, have weak BRAF inhibition activity. BRAF inhibitors have demonstrated promising efficacy in relapsed metastatic PCT after standard treatment, though they are not currently approved for this indication. *Case Presentation*. We present the case of a 48-year-old Hispanic male who initially presented with columnar-cell variant subtype of PTC and positive BRAFV600E mutation. The patient had widespread bulky metastases to lungs, chest wall, brain, and bone. *Discussion*. Initial use of vemurafenib demonstrated a 42% cytoreduction of targeted pulmonary metastases and facilitated thyroidectomy and RAI treatment. The patient achieved a durable response over 21 months in the setting of widely metastatic disease. *Conclusion*. Vemurafenib may be effectively used for cytoreduction in patients with bulky metastatic PTC to bridge them to thyroidectomy and RAI treatment.

## Introduction

Papillary thyroid carcinoma comprises more than 80% of thyroid cancers.^1^ It is highly curable with standard therapy combining thyroidectomy, neck lymph node dissection, radioactive iodine ablation, and thyrotropin suppression.^2^ Ten-year survival exceeds 85%.^3^ However, 10% to 15% of PTCs develop metastatic disease or become refractory to radioactive iodine therapy.^4^ Metastatic disease has a 10-year survival of less than 42%, and if the metastases become RAI-refractory, the survival rate is less than 10%.^5^

In PTC, aberrant and constitutive activation of the RET/PTC-RAS-RAF-MEK-MAPK-ERK kinase pathway (MAPK) leads to tumor genesis and proliferation.^6^ The classic oncogenic alterations in this pathway include activating point mutations of B-type Raf kinase (BRAF; 44%) and RAS genes (0% to 21%) as well as RET/PTC rearrangements (13% to 43%).^6,7^ The Cancer Genome Atlas whole exome sequencing project elaborates further the genomic landscape of PTC and proposes reclassification of PTC into molecular subtypes with distinct signaling and differentiating patterns for improved targeted therapies.^8^ Interestingly, BRAF mutation frequency is observed highest (~77%) in tall-cell variant PTC, which exhibits more aggressive clinical course and poorer prognosis than conventional PTC.^7^ The BRAFV600E mutation comprises more than 90% of all mutations within the kinase domain.^9^ Thus, inhibition of BRAF signaling pathway may potentially be an effective therapeutic approach.

BRAF mutation is associated with repression of thyroid-specific gene expression, particularly the sodium/iodide symporter (NIS) involved in iodine metabolism, leading to reduced RAI uptake.^10,11^ In a study by Xing et al, BRAF mutation was significantly associated with absence of ^131^I uptake in tumors and RAI resistance.^12^

Some studies show the association of BRAF mutation with aggressive clinicopathological features (advanced clinical stage, lymph node and distant metastases, extrathyroidal extension, recurrence, and poor prognosis),^12-20^ while other reports are inconclusive.^21-24^ Several molecularly targeted therapies including tyrosine kinase inhibitors (TKIs) and BRAFV600E inhibitors demonstrate promising clinical responses in BRAF-mutant PTCs. TKI have demonstrated partial responses and stable disease,^25-32^ but with minimal BRAF inhibition activity.^33^ In contrast, the BRAF inhibitor PLX4720 has demonstrated potent and selective BRAF mutation inhibition of cell proliferation.^34^ As a single agent, vemurafenib, a BRAF kinase inhibitor, has a 48% response rate and significant improved overall survival and progression-free survival with minimal side effects compared with dacarbazine in untreated metastatic melanoma.^35^ Clinical studies demonstrate that thyroid cancer with BRAFV6000E mutation responds to BRAF inhibitors such as vemurafenib.^36-38^ In phase I and II studies, BRAFV600E inhibitors yield promising clinical responses in patients with BRAF-positive RAI-resistant PTC who had prior thyroidectomy and RAI treatment.^37,39^ In our report, we describe the initial use of vemurafenib cytoreduction in a patient with widespread, bulky BRAFV600F-positive PTC to facilitate thyroidectomy and RAI treatment.

## Case Presentation

**Figure 1.**
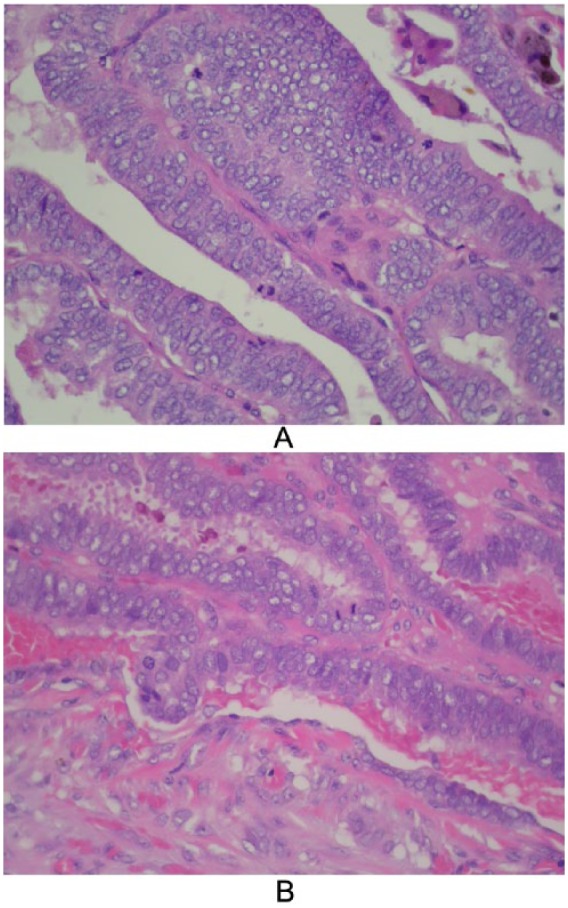
(A) Left thyroid lobe slides show papillary carcinoma with columnar cell variant characteristics (stratified nuclei, rare pseudo-inclusions, and amphophillic cytoplasm) under low magnification (400×). (B) Numerous papillae have fibrovascular cores and are lined by tall cells (height at least twice width).

In December 2013, a 48-year-old Hispanic man presented to an outside facility with a 3-month history of atypical right-sided pleuritic chest pain. Computer tomography (CT) scan showed a thyroid mass and widespread metastases to lungs, mediastinal, and supraclavicular lymph nodes. Ultrasound (US)-guided core needle biopsy of a left supraclavicular lymph node revealed PTC with morphology suggestive of columnar-cell variant (Figure 1A and B). After discharge, he did not receive any further evaluation or treatment following discharge.

A month later he presented to our facility with persistent right-sided chest wall pain, intermittent headache, and newly progressive enlargement of a tender mass in the left temple. Physical examination revealed a 3 cm palpable, tender mass at left temporal region, left submandibular swelling, and left supraclavicular lymphadenopathy. Neurological examination was unremarkable. Thyroglobulin was elevated at 2662 ng/mL, and thyroid-stimulating hormone (TSH) was 5.1 µU/mL.

**Figure 2.**
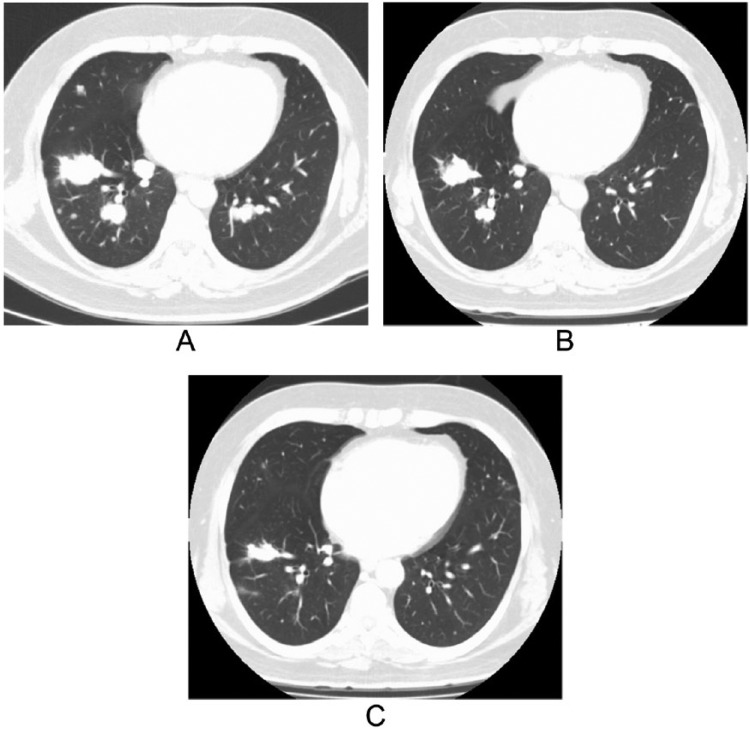
(A) Multiple bilateral pulmonary macro-metastases were presented on CT chest before starting vemurafenib. (B) After 4 months of vemurafenib, CT chest showed 42% reduction in metastatic lung nodules. (C) After first RAI and 15 months of vemurafenib therapy, CT chest showed progressive reduction in metastatic pulmonary nodules.

CT scan and magnetic resonance imaging (MRI) of the brain showed a left parietal calvarial lesion and a left occipital cerebral lesion. US of the neck revealed a 2.8 cm heavily calcified anterior isthmus thyroid nodule and extensive bilateral cervical and left supraclavicular lymphadenopathy, with the largest node measuring 2.9 cm. Chest CT demonstrated necrotic mediastinal and hilar lymphadenopathy and multiple bilateral macronodular pulmonary metastases (Figure 2A), including a 6.8 cm pleural-based mass with contiguous destruction of the right fourth rib. Repeated US-guided fine needle aspiration of a left supraclavicular lymph node confirmed the diagnosis of PTC with columnar-cell variant and a positive BRAFV600E mutation for codon 600 (c1799T>A) by pyrosequencing method.

He underwent left parietal craniectomy with complete resection of the calvarial lesion. Pathology again identified similar metastatic PTC without dedifferentiated or anaplastic component. Postoperatively, he received adjuvant radiation to left parietal surgical bed, gamma knife radiosurgery to the left occipital lesion, and palliative radiation to the symptomatic right pleural based mass. Thyrotropin suppression was initiated.

Due to the extensive bulky disease burden and bony metastases, RAI ablation (and thus thyroidectomy) was not felt feasible. Compared to conventional PTC, columnar cell variant histology portents poor prognosis.^40,41^ As a result, we opted for use of vemurafenib over sorafenib (an Food and Drug Administration–approved drug for refractory advanced differentiated thyroid carcinoma) because of its potent BRAF signaling inhibition. BRAF inhibitor demonstrated excellent response in melanoma^35^ and also yielded promising result in both anaplastic and papillary thyroid cancer harboring BRAF mutation.^36,37^ In May 2013, our patient was started on vemurafenib 960 mg twice daily.

After 4 months, he achieved a partial response with 42% reduction in the targeted pulmonary lesions (Figure 2B). Due to difficulty measuring volumetric estimates from CT images, the response was estimated using the sum products of 2 measurements of maximum diameters of a tumor from CT images that were perpendicular to each other. After 11 months, his disease burden became manageable, and we decided to proceed with a total thyroidectomy (Figure 1) and neck dissection (performed in April 2014) in order to facilitate systemic treatment with Thyrogen and RAI (received 161 millicuries [mCi] of ^131^I in April 2014). Pre-RAI’s thyroglobulin improved to 941 ng/mL, and TSH was 0.6 µU/mL. Post-RAI whole body scan (WBS) demonstrated widespread ^131^I-avid metastatic disease in suboccipital region, right neck, left sternoclavicular joint, bilateral lungs, left upper extremity, bilateral proximal femurs, pelvis, and abdomen soft tissue. CT chest also showed further reduction in pulmonary metastases (Figure 2C). Due to the possibility of impending fracture in the affected left femur, the patient did required placement of prophylactic intramedullary nail to the left femur followed by palliative radiation therapy. In October 2014, positron emission tomography CT (PET CT) showed continuing response with decrease in visceral disease. In parallel, after 19 months of vemurafenib therapy, thyroglobulin level declined from 2662 ng/mL to 474 ng/mL (TSH 0.12 µU/mL).

In February 2015, 21 months after initiating vemurafenib, PET CT showed disease progression primarily in the lower extremity osseous metastases. The patient had progress-free survival (PFS) of 21 months, which was defined as a period from the first day of treatment to the date of the first documented disease progression or date of death, whichever occurred first. Because of demonstration of disease progression and the interim Food and Drug Administration approval of lenvatinib, it was decided to stop vemurafenib, repeat Thyrogen and RAI ablation (received 208 mCi of ^131^I), and start lenvatinib. Within 2 weeks of stopping vemurafenib and before starting RAI ablation, thyroglobulin rose to 1207 ng/mL (TSH 0.06 µU/mL).

After 48 hours of Thyrogen-facilitated RAI treatment (received 208 mCi of ^131^I), he developed left eye pain, diplopia, and left facial numbness in the distribution of V1-V3 trigeminal nerve. MRI brain showed a heterogeneously enhancing hemorrhagic mass measuring 2.4 cm that involved the left trigeminal cave and cavernous sinus. Dexamethasone was started with improvement in symptoms, and he was evaluated for gamma knife radiosurgery. Post-RAI WBS demonstrated increased radioiodine uptake in the chest, left shoulder, right upper thigh, and the occipital region. However, left distal femur, right neck, abdomen, and the region of left trigeminal cave and cavernous lesion did not demonstrate further radioiodine uptake.

Our patient tolerated vemurafenib very well, and his main side effect was the development of keratosis pilaris.

## Discussion

Beyond first-line therapy with thyroidectomy and RAI ablation, there is currently no effective therapy for patients with unresectable or metastatic papillary thyroid cancer. Other studies have described vemurafenib in RAI-refractory recurrent PTC.^37-39^ This report is the first to demonstrate a durable response to vemurafenib first-line therapy in a patient with metastatic columnar cell variant subtype of PTC who was not able to undergo thyroidectomy and RAI ablation. Vemurafenib induced a durable response over 21 months in the setting of widely metastatic disease. This exceeded other reported median time to progression.^37-39^ Our PFS was also longer than those reported for TKI in phase III studies.^42,43^

The longer PFS in our patient could be due to the effect of vemurafenib to enable ^131^I uptake and resensitization with RAI ablation. However, it was unclear if our patient had RAI-resistant disease at onset. Studies have predicted repression of NIS and increased iodine metabolism in RAI-refractory metastatic disease.^10,11^ Selumetinib, a selective inhibitor of MEK1 and MEK2, and dabrafenib, a selective potent BRAFV600E inhibitor, had induced radioactive iodine uptake in a subset of patients with RAI-refractory disease and retreatment with RAI.^44,45^

Our patient had BRAF-mutated columnar cell variant history and manifested an aggressive clinical course. This was consistent with prior meta-analysis that demonstrated a significant association between BRAF mutation and aggressive clinicopathological characteristics.^13,19,20^ BRAF mutation can be useful in initial risk stratification and management of PTC. Despite high prevalence of BRAF mutations in PTC and their pivotal role in its genesis, the use of BRAF kinase inhibitor has not been widely adopted in clinical trials.

In a phase I study, vemurafenib demonstrated both stable and partial response with time to progression between 11.4 and 13.2 months in patients with PTC harboring BRAFV600E mutation who had metastasized or recurred after initial thyroidectomy and adjuvant radioactive iodine ablation.^37^ Similarly, preliminary results in a phase II study reported partial response (PR) rate of 35%, durable response rate (PR + stable disease) 58%, and median time to progression of 15.6 months in RAI-refractory patients who were TKI treatment naïve and underwent prior total thyroidectomy and RAI ablation.^39^

Other studies have explored other multitargeted kinase inhibitors. Although sorafenib targets both VEGFR and BRAF signaling, it is a relatively weak BRAF inhibitor. In phase II single arm studies with sorafenib in patients who were refractory to RAI ablation, between 15% and 31% of patients had a partial response, and approximately 34% to 74% of patients had stable disease.^26,27,29,30,32^ Thyroglobulin level was decreased by 25% to 70%, and PFS was between 12 and 18 months. A phase III study of sorafenib in RAI-resistant differentiated thyroid cancer confirmed PFS of 10.8 months versus placebo, but tumor shrinkage was observed only in 10% of patients.^42^ Randomized, double-blind, phase II trial of vandetanib, a TKI of RET, VEGFR, and EGFR signaling, in patients with refractory to radioactive iodine therapy demonstrated significant PFS of 11.1 months compared with 5.9 months in the placebo group. Again, partial response rate was much smaller compared with other studies, and stable disease was achieved in 57% of the patients.^28^

A newer TKI, lenvatinib, with similar targets to sorafenib (VEGFR, FGFR, PDGFR, RET, KIT) yielded a PFS of 18.3 months versus 3.6 months in the placebo arm in a phase III trial of patients with iodine-refractory, progressive differentiated thyroid cancers.^43^ Complete response was only 1.5%, but there was a higher partial response of 63% compared with 35% PR observed in vemurafenib. In a subgroup analysis, lenvatinib appeared to have higher activity in existing bone metastases compared with the placebo arm (23.7% vs 59% with progression of existing bone metastases). Given a higher PR, lenvatinib could have yielded better tumor debulking than vemurafenib. Unfortunately, lenvatinib was not available at the time our patient’s presentation, but this would also be a viable option to try now. Since our patient has significant bone metastases that are refractory to and progressed on RAI and vemurafenib, lenvatinib is a reasonable next therapeutic option.

In conclusion, our case report demonstrates the efficacy of a BRAF kinase inhibitor in front-line therapy in BRAFV600E-positive metastatic PTC that is not amenable for thyroidectomy and RAI ablation. Vemurafenib may be used to reduce bulky disease and to bridge patients to thyroidectomy and RAI treatment that otherwise would not be feasible.
